# Wastewater Monitoring During the COVID-19 Pandemic in the Veneto Region, Italy: Longitudinal Observational Study

**DOI:** 10.2196/58862

**Published:** 2025-01-14

**Authors:** Honoria Ocagli, Marco Zambito, Filippo Da Re, Vanessa Groppi, Marco Zampini, Alessia Terrini, Franco Rigoli, Irene Amoruso, Tatjana Baldovin, Vincenzo Baldo, Francesca Russo, Dario Gregori

**Affiliations:** 1 Unit of Biostatistics, Epidemiology and Public Health, Department of Cardio-Thoraco-Vascular Sciences and Public Health, University of Padova, Via Loredan 18, Padova, Italy, 39 049 8275384; 2 Directorate of Prevention, Food Safety and Veterinary Public Health-Veneto Region, Venice, Italy; 3 ARPAV - Agenzia Regionale per la Prevenzione e Protezione Ambientale del Veneto, U.O. Biologia, Padova, Italy; 4 Unit of Hygiene and Public Health, Department of Cardiac, Thoracic, Vascular Sciences, and Public Health, University of Padova, Padova, Italy

**Keywords:** wastewater-based epidemiology, SARS-CoV-2, COVID-19, CUSUM, WBE, cumulative sum chart

## Abstract

**Background:**

As the COVID-19 pandemic has affected populations around the world, there has been substantial interest in wastewater-based epidemiology (WBE) as a tool to monitor the spread of SARS-CoV-2. This study investigates the use of WBE to anticipate COVID-19 trends by analyzing the correlation between viral RNA concentrations in wastewater and reported COVID-19 cases in the Veneto region of Italy.

**Objective:**

We aimed to evaluate the effectiveness of the cumulative sum (CUSUM) control chart method in detecting changes in SARS-CoV-2 concentrations in wastewater and its potential as an early warning system for COVID-19 outbreaks. Additionally, we aimed to validate these findings over different time periods to ensure robustness.

**Methods:**

This study analyzed the temporal correlation between SARS-CoV-2 RNA concentrations in wastewater and COVID-19 clinical outcomes, including confirmed cases, hospitalizations, and intensive care unit (ICU) admissions, from October 2021 to August 2022 in the Veneto region, Italy. Wastewater samples were collected weekly from 10 wastewater treatment plants and analyzed using a reverse transcription–quantitative polymerase chain reaction. The CUSUM method was used to detect significant shifts in the data, with an initial analysis conducted from October 2021 to February 2022, followed by validation in a second period from February 2022 to August 2022.

**Results:**

The study found that peaks in SARS-CoV-2 RNA concentrations in wastewater consistently preceded peaks in reported COVID-19 cases by 5.2 days. Hospitalizations followed with a delay of 4.25 days, while ICU admissions exhibited a lead time of approximately 6 days. Notably, certain health care districts exhibited stronger correlations, with notable values in wastewater anticipating ICU admissions by an average of 13.5 and 9.5 days in 2 specific districts. The CUSUM charts effectively identified early changes in viral load, indicating potential outbreaks before clinical cases increased. Validation during the second period confirmed the consistency of these findings, reinforcing the robustness of the CUSUM method in this context.

**Conclusions:**

WBE, combined with the CUSUM method, offers valuable insight into the level of COVID-19 outbreaks in a community, including asymptomatic cases, thus acting as a precious early warning tool for infectious disease outbreaks with pandemic potential.

## Introduction

The COVID-19 pandemic has had a profound impact on public health, with more than 200 million confirmed cases and more than 4 million deaths worldwide as of August 2021. As the virus spread, concerns about possible transmission through wastewater became a significant public health issue [1]. Wastewater-based epidemiology (WBE) plays a pivotal role in various health-related studies. Research highlights several applications. The estimation of global nicotine consumption through WBE was reviewed in the work of Asadi et al [2], highlighting its diverse applications, shedding light on trends in nicotine consumption worldwide and the associated risks, and stressing the urgency of global action. Antibiotic-resistant bacteria are another topic of interest in WBE, as shown in the work of Tiwari et al [3], whose systematic review underscores the need for active monitoring of antibiotic-resistant bacteria, offering insights into their clinical utility, sensitivity, and uniformity. In addition, antimicrobial concentration was investigated. The systematic review by Chau et al [4] examines the concordance between estimates of the prevalence of antimicrobial resistance in wastewater and humans, while Holton et al [5] explore community-wide antimicrobial use via WBE, evaluating 16 antimicrobials and their metabolites, providing insight for improving precision in assessing drug intake. Wastewater-based antimicrobial resistance surveillance, as demonstrated in the most recent literature, appears to be an effective and promising approach to confronting such phenomena in support of public health actions. In addition, wastewater has long been useful for the surveillance of different pathogens.

Recent studies have shown that SARS-CoV-2, a COVID-19 viral strain, can be detected in wastewater samples from infected individuals, even when they are asymptomatic or have mild symptoms [1,6]. Wastewater surveillance is based on the assumption that viral RNA can be detected in the fecal matter of asymptomatic people, as the shedding of viral particles can occur before the onset of symptoms [7] and since diarrhea is a symptom reported by a significant proportion of patients with SARS-CoV-2 [8]. Furthermore, wastewater can also contain fragments of viral RNA secreted by oral or nasal routes that can enter wastewater when hands are washed. The virus can persist in wastewater for several days, although higher temperatures and lower pH levels can reduce its survival [9].

In the literature, various studies offer information on multiple aspects of COVID-19 and wastewater, including the detection of SARS-CoV-2 in wastewater [10], the persistence of the virus in wastewater [11], and the potential use of WBE as a tool to monitor the prevalence of the virus in communities [12]. The study by Ciannella et al [13] focuses on analytical procedures and epidemiological modeling in WBE for COVID-19. They highlight the lack of standardization in wastewater analytical methods, with reverse transcription–quantitative polymerase chain reactions (PCRs) being the most widely used technique for the detection and quantification of viral RNA. They suggest exploring the solid portion of wastewater due to its higher viral load and advocate the development of cost-effective portable detection devices [13]. The study by Peccia et al [14] investigates the potential of wastewater surveillance as a tool for the early detection of COVID-19 outbreaks in a community. The results show that WBE could provide early warnings of outbreaks before clinical cases are detected.

In Italy, the study by La Rosa et al [10] suggests that SARS-CoV-2 was present in northern Italy even in December 2019. The researchers analyzed wastewater samples from October 2019 to February 2020 and found positive results for the virus in multiple cities. This challenged the prior understanding that the geo-warning system primarily served to detect the virus’ presence and to monitor outbreaks before they were reported to the health care system.

In WBE, a variety of statistical methods have traditionally been employed to analyze the relationship between viral RNA concentrations in wastewater and epidemic incidence, along with other public health outcomes. Common techniques include correlation analysis, regression models [15], and principal component analysis [16], which are often used to establish associations between wastewater parameters and COVID-19 case numbers, hospitalizations, or deaths. For example, studies have frequently utilized methods such as ANOVA [17], Gaussian distribution models [18], and autoregressive integrated moving average (ARIMA) time series models to understand the temporal dynamics and predict trends in viral loads [19]. Advanced techniques include the Efficient and Practical Virus Identification System with Enhanced Sensitivity for Membrane (EPISENS-M) method, which combines highly sensitive RNA detection with mathematical modeling to predict cases of COVID-19 [20]. Additionally, the framework utilized by Dai et al [21] uses functional data analysis to detect true trends in viral concentration, incorporating known covariates such as sample storage temperature and influent volume, and employs Markov chain Monte Carlo methods to forecast future viral concentrations.

However, while these approaches are effective for retrospective analysis and prediction, they often focus on broad trend identification rather than real-time detection. Our study introduces the cumulative sum (CUSUM) chart method, a statistical process control tool commonly used in quality control [22], which offers unique advantages in WBE by detecting small shifts in viral load trends. Unlike traditional methods, CUSUM charts are particularly useful for identifying early deviations from established baselines, thus allowing earlier detection of potential outbreaks.

This study aims to investigate whether the time series of viral RNA concentrations in wastewater, expressed as genome copies (gc) per microliter, can serve as an early indicator of COVID-19–positive cases, including total positives, hospitalized cases, and those admitted to intensive care units (ICUs), in the Veneto region using CUSUM charts.

## Methods

### Overview

This study was conducted in the Veneto region of Italy, focusing on a specific timeframe during the COVID-19 pandemic. While the number of data points is limited, the study aims to provide a detailed analysis of the correlation between the viral RNA load of wastewater and the COVID-19 clinical outcomes, offering valuable information for regional public health strategies.

Our research evaluated this hypothesis by comparing the temporal trends of these case categories to SARS-CoV-2 concentrations in wastewater samples.

### Ethical Considerations

This study was conducted using publicly available, anonymized, and deidentified data, ensuring that no personal information of individuals was accessed or processed. Wastewater data were derived from environmental samples (ie, untreated wastewater) and COVID-19 epidemiological data were aggregated at the population level, preventing any direct identification of individuals. Additionally, the analysis adhered to ethical guidelines for research using secondary data, ensuring that all data were used responsibly and in accordance with the applicable privacy laws. No personal or sensitive information was collected and the study design did not involve any interventions or direct interaction with human subjects, thus exempting it from requiring formal ethical approval.

### Data Sources

This study used data from multiple sources to analyze the correlation between SARS-CoV-2 RNA concentrations in urban wastewater and COVID-19 clinical outcomes. The primary data sources included analytical data from raw wastewater samples and clinical data, including COVID-19 case data, hospitalizations, and ICU admissions, from the Presidency of the Council of Ministers’ Civil Protection Department.

These datasets were analyzed to assess temporal correlations and to identify potential early warning signals for COVID-19 surges based on wastewater monitoring.

### Wastewater Data and Study Design

The study focused on the Veneto region, selecting 10 wastewater treatment plants (WTPs) in 5 provinces, collectively serving more than 4 million inhabitants. Weekly samples were collected from October 5, 2021, to August 2, 2022.

#### Sampling Equipment and Sites

**Figure 1.**
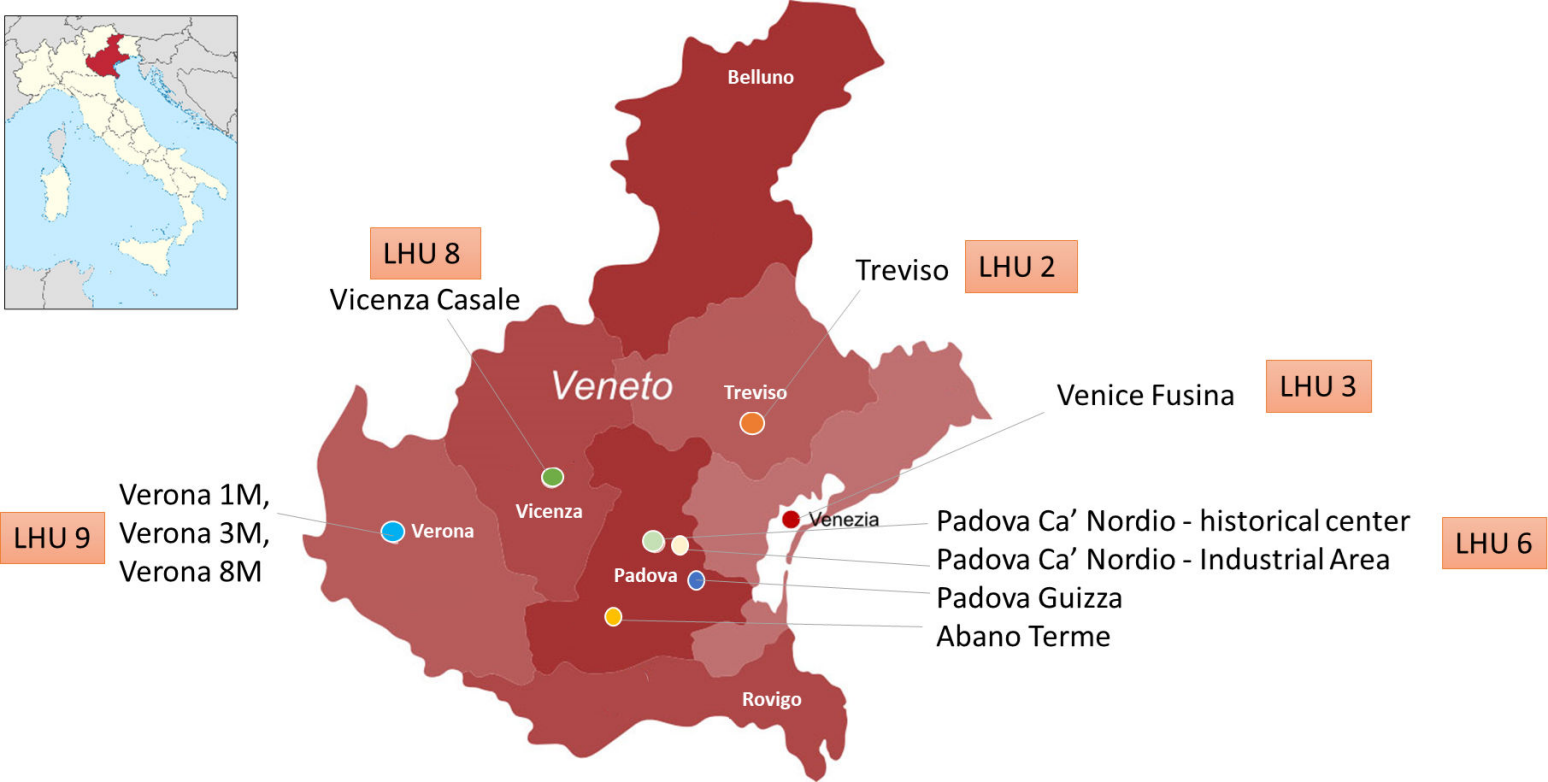
Geographic distribution of wastewater treatment plants and correspondence with LHUs in the Veneto region, Italy. LHU: local health unit.

The samples were collected using an automated sampler (compact portable sampler 6712C, ISCO) to obtain 100 mL of a 24-hour composite raw wastewater sample [10], ensuring representative capture of daily variations in wastewater composition. Each sample consisted of 20 mL subsamples of raw wastewater collected every 30 minutes for the 24-hour period. After collection, the samples were immediately refrigerated at 4 °C to preserve viral RNA integrity until analysis. Sample concentration, extraction, and viral RNA detection protocols are described in the study by Baldovin et al [23]. The selected WTPs included: Abano Terme, Padova Ca’ Nordio - historical center, Padova Ca’ Nordio - Industrial Area, Padova Guizza, Treviso, Venice Fusina, Verona 1M, Verona 3M, Verona 8M, and Vicenza Casale (Figure 1).

#### RNA Detection and Laboratory Methods

Following collection, wastewater samples were concentrated using the Epidemiological Surveillance for SARS-CoV-2 in Urban Sewage in Italy, third revision (SARI_rev3) method [24], which includes a sample concentration step involving polyethylene glycol precipitation. This process yielded a concentrated pellet from a 45 mL sample volume, which was then subjected to RNA extraction using 2 different commercial kits, following the manufacturer’s instructions. For the University of Padova samples, RNA extraction was performed using the QIAamp viral RNA mini kit (Qiagen). The samples were collected at the following locations: Abano Terme, Padova Ca’ Nordio - historical center, Padova Ca’ Nordio - Industrial Area, and Padova Guizza. For the Regional Agency for Environmental Protection and Prevention of Veneto samples, RNA extraction was conducted using the eGENE-UP system (Biomerieux), a well-established method for SARS-CoV-2 RNA recovery. These samples were collected from Treviso, Venice Fusina, Verona 1M, Verona 3M, Verona 8M, and Vicenza Casale. For all samples, RNA was treated with the OneStep PCR Inhibitor Removal Kit (Zymo Research) after extraction to remove PCR inhibitors. The final volume of the RNA elution was standardized to 100 µL.

Reverse transcription–quantitative PCR assays targeting the SARS-CoV-2 open reading frames 1ab (ORF1ab) (nonstructural protein 14) gene were used for quantification. A limit of detection was established at 2 gc/µL. For measurements below this threshold, the values were recorded as half of the limit of detection to account for the presence of the low level. PCR inhibition was routinely assessed using an internal Mengovirus control and recovery efficiency was confirmed for each sample using this control as well.

#### Replicates and Quality Control

Each wastewater sample was processed in duplicate to ensure the reproducibility and accuracy of viral load quantification. Additionally, a spiking experiment was performed periodically using known concentrations of synthetic SARS-CoV-2 RNA to verify the precision of the assay on different matrices. This approach enabled the detection of any potential assay failures or matrix effects that could impact quantification. To account for the variability in influent flow rates and population size, viral load data were normalized per liter of wastewater and per capita. This normalization facilitated comparison across different WTPs and over time. Detailed records of each WTP’s daily influent volume, as well as specific operational characteristics, were maintained and integrated into the data analysis to ensure that any observed variations in viral concentrations could be accurately interpreted.

### Positive COVID-19 Case Data

The Presidency of the Council of Ministers’ Civil Protection Department diligently managed, processed, and provided data on active and cumulative confirmed COVID-19 cases and daily COVID-19-related deaths for public use under the Creative Commons Attribution 4.0. The population of confirmed COVID-19 cases in the area served by each WTP was identified based on local health units (LHUs) as follows: LHU 2 (Treviso collector); LHU 3 (Venice Fusina collector); LHU 6 (Abano Terme, Padova Ca’ Nordio - historical center, Padova Ca’ Nordio - Industrial Area, and Padova Guizza collectors); LHU 8 (Vicenza Casale collector); and LHU 9 (Verona 1M, 3M, and 8M collectors) (Figure 1).

### Statistical Analysis

Continuous variables were reported as median and first and third quartiles, and categorical variables as percentages and absolute numbers. The trend of wastewater was reported as an absolute value for each measurement, and the trend in the number of positive cases was shown as positives per province population multiplied by 100,000.

#### CUSUM Charts

To compare COVID-19 cases and trends in wastewater, we used CUSUM charts. Originally developed by Page in 1954 [25], CUSUM charts have since been widely adopted across various fields, including health care, for quality control purposes. The key advantage of a CUSUM chart lies in its cumulative nature, where each plotted point reflects not only the current value but also the cumulative effect of all previous data points [22]. This makes CUSUM charts especially sensitive to small deviations that could indicate a gradual trend change, positive or negative, within a dataset [26].

This graphical tool detects slight deviations from a target value (in this case, the mean) by considering current and previous values in the series. Specifically, the CUSUM chart is based on a statistic derived from the standard deviations of the process, assuming that the empirical distribution follows a normal distribution with a mean of zero. Statistic values that exceed the upper and lower limits (typically set at 4σ, where σ represents the standard deviation of the process) are marked as “out of control” points. These points can be interpreted as “structural breakpoints” or “turning points,” indicating significant shifts in the series’ trend, either positive or negative. The “differential” signifies the difference between the time (“t”) and the value at the values of time (“t-1”). The CUSUM charts for positive case differentials employ upper and lower limits at 4σ, while those for gc/µL concentrations use a threshold at ±2.782 [27] due to lower data variability (varpositive=16,048,128; varRNA=3070.525 gc/µL).

The analyses were performed using R [28], with the *qcc* package used for the CUSUM chart [29]; *ggplot* [30] and *ggplot2* [31] were used to generate the graphs.

## Results

### Overview

Table 1 provides an overview of the WTPs considered during the sampling period. It offers information on their key characteristics, including flow rates (m^3^/24 h) and total suspended solids concentrations (mg/L). These parameters exhibit variations at different locations, indicating the diverse compositions of the wastewater. The table also highlights the volume of collected samples (mL) and the corresponding distribution across different sample sizes. The concentrations of total suspended solids showed diversity, ranging from 94.25 mg/L in Treviso to 207.00 mg/L in Verona 8M.

**Figure 2.**
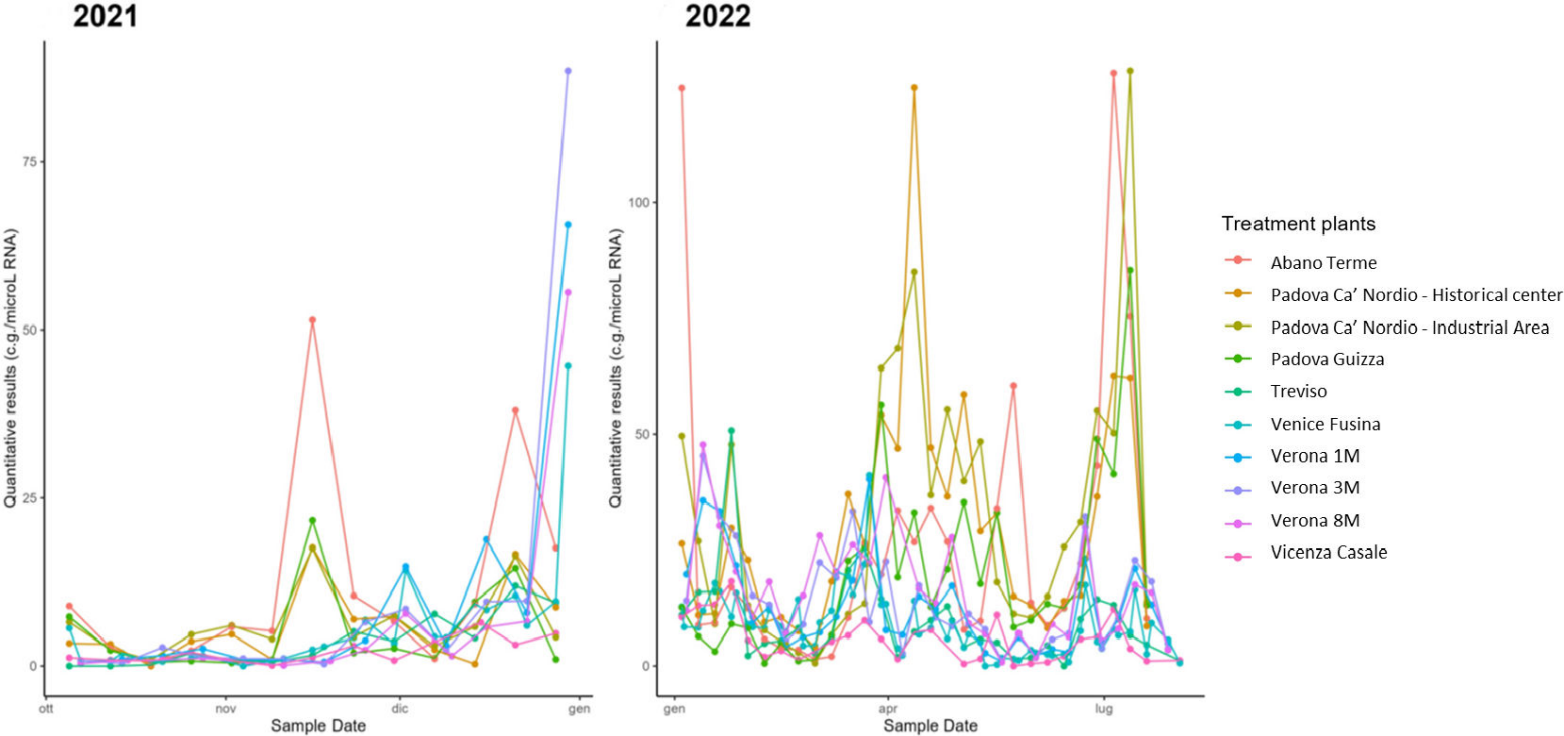
Temporal trends of SARS-CoV-2 RNA concentrations in wastewater throughout the Veneto region, Italy, during 2021 and 2022.

Figure 2 shows the trend of wastewater for 2021 and 2022, divided by the different collectors. It should be noted that there are different peaks in wastewater load during specific periods, including November 2021, late December 2021, and January 2022, followed by recurring peaks in April and July 2022.

**Table 1.** Descriptive characteristics of wastewater treatment plants in the Veneto region, Italy, considered in the study. Continuous variables are reported as I, II, and III quartiles, whereas categorical variables are reported as percentages and absolute numbers.

	Flow rates (m^3^/24 h), I/II/III quartile	Total suspended solids (mg/L), I/II/III quartile	Sample volume (125 mL), n (%)	Sample volume (250 mL), n (%)	Sample volume (500 mL), n (%)
Abano Terme (n=44)	4927.00/5428.50/6059.50	170.00/232.00/276.00	0 (0)	0 (0)	44 (100)
Padova Ca’ Nordio - historical center (n=44)	22,074.25/24,677.00/27,760.75	112.00/172.00/234.00	0 (0)	0 (0)	44 (100)
Padova Ca’ Nordio - Industrial Area (n=44)	21,626.75/24,677.00/27,760.75	112.00/170.00/235.00	0 (0)	0 (0)	44 (100)
Padova Guizza (n=44)	3087.25/3275.50/3406.25	16.00/47.00/74.50	0 (0)	0 (0)	44 (100)
Treviso (n=44)	13,477.50/14,365.00/14,830.00	94.25/123.50/169.75	0 (0)	0 (0)	44 (100)
Venice Fusina (n=87)	72,906.50/87,562.00/100,155.25	68.50/116.00/193.00	34 (39)	51 (59)	2 (2)
Verona 1M (n=43)	12,657.50/12,874.00/13,089.50	117.00/181.00/230.00	0 (0)	0 (0)	43 (100)
Verona 3M (n=43)	11,232.00/14,688.00/17,237.00	132.50/183.00/203.50	0 (0)	0 (0)	43 (100)
Verona 8M (n=43)	33,739.50/34,474.00/39,571.00	207.00/231.00/265.00	0 (0)	0 (0)	43 (100)
Vicenza Casale (n=44)	27,052.75/30,018.50/32,466.75	85.75/101.00/119.25	0 (0)	0 (0)	44 (100)

### CUSUM Chart Results in the First Period

Daily total COVID-19–positive cases, hospitalized cases, and admissions to the ICU were compared with daily viral concentrations. The analyses considered recovery efficiency (percentage recovery value), which was used as a weighting factor. The results obtained, particularly with respect to the peak concentrations, remained consistent even after adjustment for recovery values.

**Figure 3.**
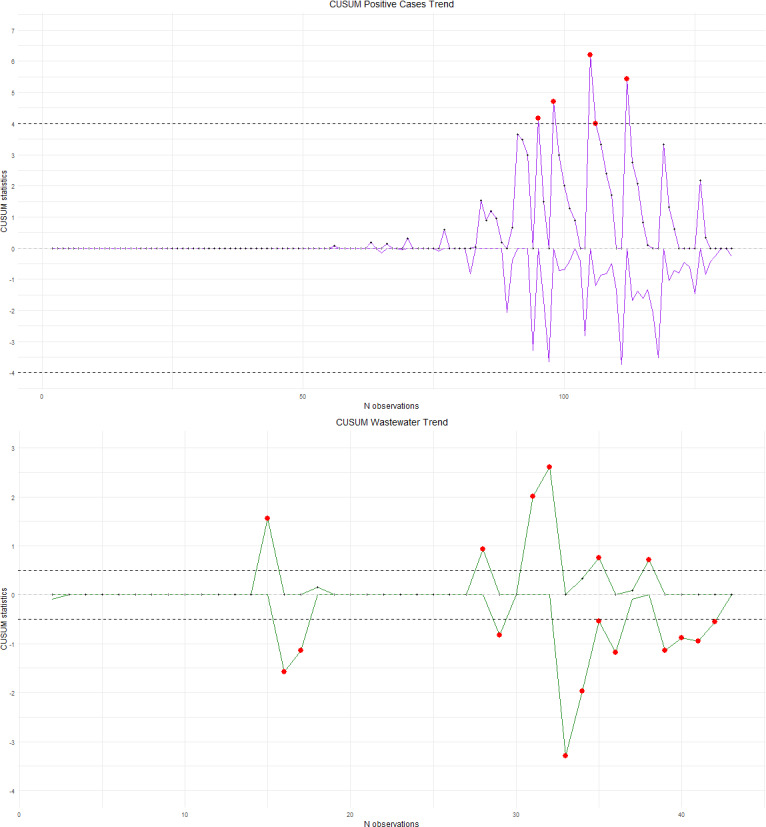
CUSUM analysis of COVID-19–positive cases (top) and SARS-CoV-2 RNA detected in wastewater (bottom) in the Veneto region, covering the period from October 5, 2021, to February 14, 2022. CUSUM: cumulative sum.

Figure 3 illustrates the CUSUM charts that show the interaction between positive cases and wastewater trends, plotting progressive observations on the x-axis, while the y-axis shows the cumulative deviation from the target, highlighting shifts and trends over time. Notable spikes indicate significant increases, suggesting potential outbreaks or changes in viral concentration.

In Figure 3, we observe a significant positive differential value beyond the upper limit at the 106th data point in the CUSUM Positive Cases Trend chart, which corresponds to the observation made on January 19, 2022. This indicates a substantial increase in positive cases. When comparing this with the CUSUM Wastewater Trend chart, several positive and negative peaks appear. In particular, there is a peak in wastewater effluent between December 30, 2021, and January 25, 2022, and each of these effluent peaks aligns with a subsequent peak in positive case differentials between January 3 and 31, 2022.

Table 2 presents data on the correspondence between significant peaks in wastewater SARS-CoV-2 RNA concentrations and subsequent peaks in COVID-19 cases, distinguishing between total cases, hospitalized cases, and admissions to the ICU.

**Table 2.** Temporal correspondence between peaks in SARS-CoV-2 RNA concentrations in wastewater and COVID-19 cases in the Veneto region (October 5, 2021, to February 14, 2022).

Dates of wastewater peaks	SARS-CoV-2 RNA concentration, gc/µL	Dates of COVID-19 peak cases	COVID-19 cases, n	Difference in days, n
Total positive COVID-19 cases				
12/21/2021	100.75	12/27/2021	8883	6
12/30/2021	188.73	1/03/2022	16,848	4
1/04/2022	247.79	1/10/2022	22,024	6
1/13/2022	140.62	1/17/2022	25,283	4
1/25/2022	172.71	1/31/2022	15,372	6
Hospitalized COVID-19 cases				
12/21/2021	100.75	12/27/2021	284	6
12/30/2021	188.73	1/03/2022	265	4
1/04/2022	247.79	1/07/2022	253	3
1/13/2022	140.62	1/17/2022	254	4
Hospitalized in intensive care units COVID-19 cases				
12/21/2021	100.75	12/27/2021	18	6

For example, for total positive cases, it should be noted that effluent peaks on December 30, 2021, and January 4, 2022, were followed by significant increases in positive cases, with a delay of 4 to 6 days. This suggests a correlation between wastewater changes and surges in total cases of COVID-19.

Analysis of the CUSUM charts reveals that effluent concentration peaks consistently preceded positive case peaks by an average of 5.2 days for total positive cases. In the case of hospitalized patients, the average time lag between the detection of a significant effluent value and the subsequent detection of a significant value in hospitalizations was 4.25 days.

Similarly, for positive cases that required hospitalization, effluent peaks on December 30, 2021, and January 4, 2022, were followed by case peaks, although with a slightly shorter delay of 3 to 4 days. This close temporal relationship between effluent peaks and hospitalizations is a significant finding.

Furthermore, data indicate that effluent peaks on December 21 and 27, 2021, coincided with the increase in ICU admissions 6 days later, underlining a consistent trend in which wastewater changes anticipated changes in the need for critical care.

### CUSUM Chart Results in the Second Period

**Figure 4.**
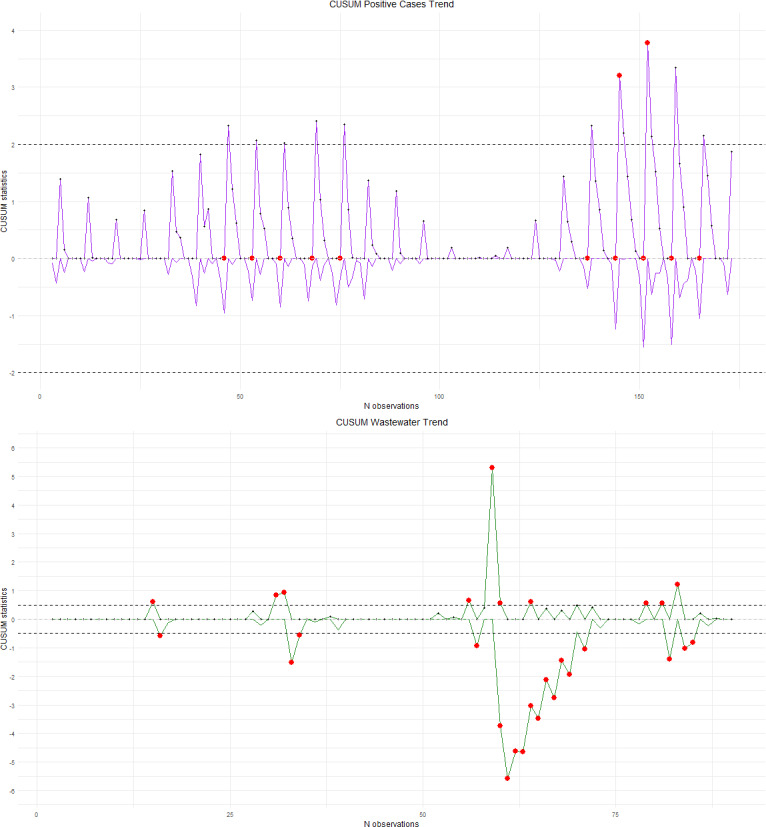
CUSUM analysis of positive cases for COVID-19 (top) and SARS-CoV-2 RNA trends in wastewater (bottom) in the Veneto region, Italy, February 14, 2022, to August 2, 2022. CUSUM: cumulative sum.

To validate the hypotheses and results, the same analysis was performed on the series of positive cases within the time frame from January 14 to August 2, 2022, (Figure 4). Furthermore, Figure 4 shows the complete series of the differential of viral load in wastewater effluent.

As shown in Table 3, seven turning points in the effluent series corresponded to turning points in the positive case series. The average number of days between a wastewater peak and a clinically confirmed case peak was 6. In particular, a peak in the effluent dated April 7, 2022, was associated with an elevated positive case count, although it did not differ significantly from other outbreak values.

The results of the comparison of the CUSUM charts for total positive cases and effluents until August 2, 2022, align with the findings observed in the analysis up to February 13, 2022. The average number of days between an effluent peak and a positive case peak was 6.

**Table 3.** Temporal correspondence between peaks in SARS-CoV-2 RNA concentrations in wastewater and COVID-19 cases in the Veneto region (February 14, 2022, to August 2, 2022).

Dates of wastewater peaks	SARS-CoV-2 RNA concentration, gc/µL	Dates of COVID-19 peak cases	Total positive COVID-19 cases, n	Difference in days, n
3/29/2022	227.96	4/05/2022	8205	7
4/07/2022	715.86	4/12/2022	7854	5
4/12/2022	290.86	4/20/2022	8922	8
4/26/2022	158.38	4/27/2022	8814	1
6/21/2022	109.60	6/28/2022	8753	7
6/28/2022	209.81	7/05/2022	10,884	7
7/05/2022	189.48	7/12/2022	12,571	7

## Discussion

### Principal findings

This study provides valuable information on the use of WBE as an early warning system for COVID-19 outbreaks in the Veneto region of Italy. Using CUSUM charts, we were able to detect significant changes in viral RNA concentrations in wastewater and assess their correlation with COVID-19 clinical outcomes, such as positive cases, hospitalizations, and admissions to the ICU.

Our findings confirm the potential of WBE to anticipate COVID-19 trends, with viral peaks in wastewater consistently preceding peaks in reported cases by an average of 5.2 days. Similarly, wastewater data provided early indications of increases in hospitalizations and admissions to the ICU, with a lag time of 4.25 and 6 days, respectively. These lead times are crucial for public health preparedness, allowing authorities to mobilize resources and implement containment measures before the number of clinical cases surges.

Our results align with previous WBE studies for the monitoring of SARS-CoV-2. Henriques et al [32], in their 2023 study, underscore the importance of WBE in assessing the presence and prevalence of SARS-CoV-2, particularly in regions with limited clinical data. WBE is a crucial complementary tool for the early detection and surveillance of COVID-19 outbreaks. It helps to estimate clinical cases and evaluate the effectiveness of vaccination programs [32].

The systematic review and meta-analysis by Li et al [33] emphasize the varying correlation between SARS-CoV-2 RNA concentrations in wastewater and clinically confirmed cases of COVID-19. Environmental factors, WBE sampling designs, and epidemiological conditions influence the strength of this correlation. Their study underscores the need to understand the dynamics of viral shedding, in-sewer decay, and sampling strategies to accurately estimate COVID-19 cases through WBE [33]. Furthermore, a study by Sodhi and Singh in 2022 [34] explores SARS-CoV-2 in wastewater, highlighting the presence of the virus even before symptoms. They advocate revised guidelines and advanced viral remediation techniques.

The temporal correlations between wastewater trends and clinical outcomes underscore the potential usefulness of WBE as a supplementary tool for public health surveillance. However, our analysis revealed a lead time of only 5 days when considering COVID-19–positive cases. Although a 5-day warning can help prepare health care resources, it comes with certain limitations. Relatively short lead times can lead to a faster but less reliable response, especially given the complexities of reorganizing health care resources and managing intensive care. Therefore, while we acknowledge the value of short-term alerts, it is crucial to strike a balance between timeliness and reliability when implementing preventive and resource management measures. To better focus on the increase in health care resources, it is essential to obtain more data on hospitalized cases and admissions to the ICU for COVID-19. However, during the considered period, these data are not robust enough to suggest a mean number of days to prepare for an increase in the number of beds for admitted patients.

The analysis in Table 2 shows a 13-day gap between the peak in the SARS-CoV-2 RNA concentration in wastewater on January 4, 2022, and the peak in reported cases of COVID-19 on January 17, 2022. This longer-than-expected delay highlights the variability inherent in WBE. Several factors could explain this discrepancy, including complex disease dynamics, inconsistent lag times due to varying public health responses, and potential delays in testing and reporting during periods of high case numbers. These factors underscore the need for careful interpretation of the correlations between wastewater data and clinical case trends, as the relationship may not always be linear or immediate.

The comparison between the fall of 2021 and the spring of 2022 reveals a potential gap in clinical surveillance, identified through the analysis of divergent trends between wastewater concentrations and reported cases of COVID-19. Specifically, while wastewater data indicated fluctuations in viral RNA concentrations, the number of reported COVID-19 clinical cases began to decline from April 2022, suggesting either a decrease in testing or reporting accuracy or a genuine decrease in cases not fully captured by the wastewater analysis. This divergence highlights the importance of integrating multiple data sources, including wastewater and clinical data, to provide a comprehensive overview of disease trends.

Despite current challenges, future research aims to improve sampling and analysis protocols, understand viral dynamics in sewer systems, and exploit artificial intelligence and big data to better monitor wastewater. The study by Nayak et al [18] highlights the potential of machine learning, particularly in diverse sewer sheds, using time series models such as long short-term memory and various data parameters for accurate trend forecasting. In parallel, the work by Jiang et al [35] underscores the crucial role of WBE in tracking COVID-19 transmission.

In the field of WBE, several methodologies have been developed to predict future COVID-19 cases, including our method, the EPISENS-M technique of Ando et al, and the statistical framework proposed by Dai et al. Although all 3 methods share the objective of using wastewater data to monitor and forecast the evolution of the pandemic, they differ significantly in their approach, robustness, and applicability.

The EPISENS-M method, developed by Ando et al [20], represents a significant advancement in the sensitive detection of SARS-CoV-2 RNA in wastewater. This method utilizes membrane adsorption and direct RNA extraction to enhance detection sensitivity, allowing for accurate prediction of clinical cases even in low-prevalence settings. However, a key limitation of this approach is its reliance on recent clinical data for calibration, which may reduce its effectiveness in situations where such data are not readily available.

On the other hand, Dai et al [21] developed a sophisticated statistical framework based on Bayesian models and functional principal component analysis to address the challenges posed by noisy and sparsely sampled data. Although their approach is extremely useful for interpreting viral concentration data in wastewater and mitigating fluctuations due to technical errors, it is not designed to directly predict clinical cases of COVID-19. This limits its applicability as a direct epidemiological forecasting tool.

The use of the CUSUM method in this study marks a novel approach to identifying subtle shifts in SARS-CoV-2 trends, offering a more sensitive detection of deviations from established baselines. Unlike EPISENS-M, our method is less dependent on recent clinical data, making it potentially more robust in contexts with limited clinical monitoring. Furthermore, compared to the method of Dai et al, our approach focuses specifically on epidemiological prediction, providing a direct advantage for public health planning.

### Limitations

Despite its potential, our study has limitations. The reliance on a single region and a specific time frame restricts the generalizability of our findings.

Furthermore, the significant heterogeneity observed in flow rates, suspended solid concentrations, and volume distribution provides information on the differences in the sites considered. The sites reflect the data on specific cities; so, while we have the number of positive cases per province, we need to exercise caution when generalizing these findings to the whole region. Additionally, the study’s reliance on SARS-CoV-2 RNA concentrations as an early indicator may overlook other potential contributors to wastewater dynamics and require further validation through complementary approaches.

This study focused on the correlation between SARS-CoV-2 RNA concentrations in wastewater and clinical indicators of COVID-19, without accounting for the dynamics of population mobility or additional biomarkers in wastewater. While these factors can influence wastewater analysis and provide deeper insights, they were beyond the scope of the current study. Future research could integrate mobility data and additional biomarkers to improve the predictive capacity of WBE.

Validation analyses were performed only for positive cases due to a low number of peaks for hospitalized and ICU cases. Therefore, the study acknowledges the need for further validation and standardization of analytical methods in wastewater analysis. Additionally, while the study highlights correlations, the cause cannot be definitively established. Future research could explore the generalizability of these findings in different regions and populations.

### Conclusions

This study confirms that WBE, even when applied at a regional level with limited data points, can provide early warnings for COVID-19 outbreaks. The use of CUSUM charts enabled the sensitive and timely detection of shifts in viral load trends, offering crucial lead times for public health interventions. Our findings highlight the value of integrating WBE into public health surveillance systems, especially in areas where clinical data may not be immediately available.

The results confirm the reliability of wastewater analysis in anticipating COVID-19 trends, particularly at the LHU level, which is vital for informed public health planning and proactive responses. The potential of WBE to monitor the spread of SARS-CoV-2, including emerging variants, underscores the importance of continued efforts to improve wastewater monitoring and treatment.

More research is essential to refine the detection and quantification methods for pathogens and viruses in wastewater, as well as to evaluate their feasibility in different contexts. Ultimately, this approach offers a robust and valuable tool for epidemiological surveillance, contributing significantly to the early detection and management of infectious disease outbreaks with pandemic potential.

## Abbreviations

ARIMA: autoregressive integrated moving average
CUSUM: cumulative sum
EPISENS-M: Efficient and Practical Virus Identification System with Enhanced Sensitivity for Membrane
gc: genome copies
ICU: intensive care unit
LHU: local health unit
ORF1ab: open reading frames 1ab
PCR: polymerase chain reaction
WBE: wastewater-based epidemiology
WTP: wastewater treatment plant
